# Health related QoL in celiac disease patients in Slovenia

**DOI:** 10.1186/s12955-020-01612-9

**Published:** 2020-11-04

**Authors:** Eva Turk, Dušanka Mičetić-Turk, Maja Šikić-Pogačar, Alojz Tapajner, Veljko Vlaisavljević, Valentina Prevolnik Rupel

**Affiliations:** 1 Science Centre Health and Technology, University of South-Eastern Norway, Grønland 53, 3045 Drammen, Norway; 2 Faculty of Medicine, Department of Pediatrics, University of Maribor, Taborska ulica 8, 2000 Maribor, Slovenia; 3 IVF Adria Consulting, Ljubljanska ul. 9, 2000 Maribor, Slovenia; 4 Institute for Economic Research, Kardeljeva ploščad 17, 1000 Ljubljana, Slovenia

**Keywords:** Health related quality of life, HRQoL, Celiac disease, EQ-5D

## Abstract

**Background:**

Measurements of health-related quality of life (HRQoL) among celiac disease patients using a validated questionnaire have been lacking in Slovenia. This study aims to measure HRQoL in celiac disease (CD) patients using EQ-5D internationally validated questionnaire and comparing it to the HRQoL of the general population.

**Methods:**

In this cross sectional analysis all of the approximately 2000 members of the Slovenian Celiac Society were invited to take part. We used a 3 step approach for recruitment and data collection. HRQoL was evaluated through the EuroQoL EQ-5D-5L instrument (Slovenian version) and analysed using the ordinal logistic regression.

**Results:**

Out of 321 patients who gave their consent, 247 celiac patients were included in the study (77%). 68% of the participants were female and 53% of them lived in an urban setting. Most patients originated from North-East Slovenia, whereas approximately 30% of patients came from other Slovenian regions. The EQ-5D respondents’ self-reported health status at the time of the study show that most patients have slight or no problems when living with CD. The duration of the gluten-free diet, academic education and rare (< 1 × year) doctor visits affect EQ-5D in a positive way. On the other hand, higher age and chronic rheumatic disease were negatively associated with EQ-5D also when compared to the general population.

**Conclusion:**

This is the first Slovenian study to measure the HRQoL of Slovenian CD patients, using an internationally validated questionnaire. The results of our study show that HRQoL is slightly impaired among Slovenian patients with CD. Clinical characteristics are better determinants of their HRQoL than socio-demographic factors. Greater awareness of the impact of CD on patients’ HRQoL would improve the holistic management of CD patients.

## Introduction

Celiac disease (CD) is an immune-mediated systemic disorder activated by the ingestion of gluten and related prolamins in genetically susceptible individuals. In the past, CD was considered a gastrointestinal disease of childhood; today, CD is recognized as a systemic disease. With a prevalence of approximately 1%, it is one of the most frequent chronic diseases in Europe [1–3]. Slovenia does not differ significantly from other European Union countries with regard to the prevalence of CD [4]. In the past 2 decades, a growing interest in the study and evaluation of the health-related quality of life (HRQoL) of patients with chronic diseases has been noted. HRQoL is a broad multidimensional concept that usually includes self-reported measures of physical and mental health. HRQoL refers to an individual’s overall well-being and daily functioning. It can be divided into three principal components: physical health, which addresses individual’s daily functioning, pain, physical disability; mental health, addressing mood, self-esteem, perception of well-being, perceived stigma; and social health, addressing social activities and relationships [5]. In addition to objective clinical and laboratory parameters, when evaluating the state of health and success of treatment, it is extremely important to include patient’s personal experiencing of the disease and the effect it has on the quality of his/her life. In patients with CD, numerous factors may adversely affect HRQoL. These factors are the stigma of a chronic disorder, poor adherence to gluten-free diet (GFD), social and economic issues associated with lifelong GFD, associated medical comorbidities, as well as intestinal and extra-intestinal complications in CD increase and the self-perceived burden of illness [6]. The term HRQoL derives from the well-known and broad definition of health as given by the World Health Organization (WHO), which defines health as a condition of complete physical, mental and social well-being and not merely as the absence of disease or disability. HRQoL is the product of psychological, physical, and social well-being, and the perception of one’s position in life compared to others [7]. Evidence on patients’ HRQoL can be obtained using patient-reported outcome (PRO) measures. Both generic (general) and individual (specific) tools can be used to measure the HRQoL in CD [8, 9]. Monitoring HRQoL enables a more comprehensive evaluation of the disease and benefits of treatment. In most countries, the majority of the previous studies used generic tools (SF-36 or SF-12), but not EQ-5D.

In Slovenia, there is a lack of literature on HRQoL and symptoms experienced by celiac patients. Studies, which evaluated HRQoL of patients with CD were conducted without validated questionnaires. Therefore, the objective of the current study was to measure HRQoL in the Slovenian CD patients using a validated general HRQoL questionnaire (EQ-5D) and to assess its relationships with sociodemographic and health factors.

## Methods and data

This cross-sectional study was conducted between August and December 2017. Multiple questionnaires were used to collect the information in relation to demographics, CD and HRQoL. Duration of CD and comorbidities were extracted from the health records. All of the approximately 2000 members of the Slovenian Celiac Disease Society (SCDS) were invited to take part in the study. Included were members of the SCDS aged between 18 and 55. To become a member of SCDS, a confirmed CD diagnosis by biopsy is the requirement. The members were addressed by the society’s management. They collected informed consent form from members interested to participate. About 321 society members agreed to participate by signing the consent form and stating their home address. There were three steps taken in data collection: 1. the participants sent the informed consent form to one of the authors’ address by the regular post; 2. In the next step questionnaires were sent to the participants. 3. In the final step, we obtained 297 questionnaires by regular post, 40 of them were excluded due to missing data. The final sample size included 247 Slovenian celiac patients.

After obtaining a signed informed consent form, all participants were administered the same standardized questionnaire. Participants also gave their consent to extract data from their health records regarding duration of CD and comorbidities. The authors collected data and guaranteed anonymity and confidentiality of participants and their data. The current study was part of a national project (ref: Slovenian Research Agency, Code: J3-7177), which was approved by the National Medical Ethics Committee on 29 November 2014 (No. 49/09/14).

HRQoL was evaluated through the EuroQoL EQ-5D-5L instrument (Slovenian version) [10]. EQ-5D is a standardized measure of health status developed by EuroQol Group to provide a simple, generic measure of health for clinical and economic appraisal [11]. It is a short self-administered generic utility measure that provides both a descriptive profile and an overall index for HRQoL. The questionnaire is a self-reported description of the subject’s current health in 5 dimensions i.e., mobility, self-care, usual activities, pain/discomfort and anxiety/depression. The subjects are asked to value their own current level of health in each dimension into one of five levels of health (no problems, slight problems, moderate problems, severe problems, extreme problems). The combination of these alongside the conditions “death” and “unconscious” enables description of 3127 different health states. Each health state can be ranked and transformed into a single score called the utility or index value [12]. Slovenian 3L value set is based on a representative sample of 225 members of the Slovene general population using the EQ-5D-3L questionnaire [13]. The 3L values obtained in Slovenian study were transformed into 5L values using the mapping algorithm [14] and were applied to the own health states of the CD patients.

### Statistical analysis

Descriptive statistics were used to characterize patient demographics and clinical details. Continuous variables were reported in terms of mean values, and categorical variables as numbers and percentages. The sample data was described by frequencies (%), mean (SD) or by median value for EQ-5D score. Variability around mean values was measured by standard deviations, while precision around mean values and/or mean differences of key variables was reported using 95% confidence intervals. Only patients with complete data on all measured variables were included in the analyses. The EQ-5D scores were classified as ordinal measurement scales as suggested by Alava et al. [15]. Ordinal logistic regression was used to identify associations between EQ-5D, patient characteristics, comorbidities and duration of gluten free diet. Results are presented as an odds ratio with 95% confidence interval and p-value. For the purposes of logistic regression to reduce the number of ordinal categories, the EQ-5D score was collapsed into 6 levels (1.0, 0.9, 0.8, 0.7, 0.6, 0.5 or lower). Statistical analysis was performed using IBM SPSS software, version 25.0 (IBM Corp., Armonk, NY). Statistical significance was set at *p* < 0.05.

## Results

Out of 321 patients who gave their consent, 247 celiac patients were included in the study (77%). As shown in Table 1, 68% of the participants were female and 53% of them lived in an urban setting. Two thirds of them were married or lived in a civil union and a third of the participants did not have children.

**Table 1:** Sample description

	n = 247	%
Sex		
Male	79	32.0
Female	168	68.0
Education		
High school or less	97	39.3
College	131	53.0
University	19	7.7
Marital status		
Married or civil union	184	74.5
Single	63	25.5
Children		
None	83	33.6
One	52	21.1
Two	80	32.4
Three or more	32	13.0
Place of living		
Urban	131	53.0
Rural	116	47.0
Smoking status		
No	214	86.6
Yes	33	13.4
Violation of the diet		
No	204	82.6
Yes	43	17.4
Prescription drugs		
No	161	65.2
Yes	86	34.8
Visits to general practitioner		
Once a month or more	12	4.9
More than once a year	140	56.7
Once a year or less	95	38.5
Other diseases		
Diabetes mellitus type 1	6	2.4
Thyroid diseases	36	14.6
Chronic rheumatic disease	29	11.7
Dermatitis	14	5.7
Age (years)	43.2 ± 7.1	25–77
Duration of gluten free diet (GFD) (years)	15.8 ± 6.4	1–42

Most patients originated from North-East Slovenia, whereas approximately 30% of patients came from other Slovenian regions. For the group of adult CD patients who participated in the study, the diagnosis was made on the basis of clinical symptoms, histological examination of the small intestinal mucosa, and serological tests.

EQ-5D-5L scores were in range from 0.379 to 1.000, median was 0.885, interquartile range was 0.725–1.000.

Associations between patient characteristics and EQ-5D-5L score are presented in Table 2. The longer duration of GFD (OR = 1.04, 95% CI = 1.02–1.08, *p* = 0.036) academic education (OR = 8.42; 95% CI = 1.72–39.11, *p* = 0.032) and rare (once a year or less) visits to general practitioner (OR = 6.91, 95% CI = 1.87–25.21, *p* = 0.019) were positively associated with EQ-5D.

**Table 2:** Multivariable associations between patient characteristics and the EQ-5D-5L score

	OR	95% CI		*p*
Age (years)	0.94	0.91	0.97	< 0.001
Duration of gluten free diet (years)	1.04	1.02	1.08	0.036
Sex				
Male	1.00			
Female	0.94	0.35	2.56	0.909
Education				
High school or less	1.00			
College	1.46	0.72	3.00	0.298
University	8.42	1.72	39.11	0.032
Place of living				
Urban	1.00			
Rural	0.70	0.35	1.39	0.306
Marital status				
Married or civil union	1.00			
Single	0.63	0.24	1.65	0.352
Children				
None	1.00			
One	1.66	0.51	5.43	0.400
Two	0.86	0.27	2.73	0.801
Three or more	0.43	0.12	1.54	0.193
Occasional violation of gluten free diet				
No	1.00			
Yes	0.99	0.40	2.47	0.983
Diabetes type 2				
No	1.00			
Yes	1.32	0.37	4.64	0.561
Thyroid disease				
No	1.00			
Yes	0.67	0.23	1.92	0.452
Chronic rheumatic disease				
No	1.00			
Yes	0.23	0.08	0.65	0.006
Dermatitis				
No	1.00			
Yes	0.68	0.18	2.65	0.581
Prescription drugs				
No	1.00			
Yes	0.68	0.30	1.53	0.348
Visits to general practitioner				
Once a month or more	1.00			
More than once a year	3.54	0.79	15.94	0.099
Once a year or less	6.91	1.87	25.21	0.019

Nagelkerke R^2^ = 0.364

*OR* odds ratio, *95% CI* 95% confidence interval

On the other hand, higher age (OR = 0.94, 95% CI = 0.91–0.97, *p* < 0.001) and chronic rheumatic disease (OR = 0.23, 95% CI = 0.08–0.65, *p* = 0.006) were negatively associated with EQ-5D. Explanatory variables included in the multivariable regression model explained 36.4% of the variation in EQ-5D (Nagelkerke R^2^ = 0.364, χ^2^ = 56.111, *df* = 18, *p* < 0.001).

The differences in self-reported health status at the time of the study between general population and CD patients are presented in Table 3. The results demonstrate that most patients have slight or no problems when living with CD. None of the CD patients reported any extreme problems in any of the EQ-5D-5L categories. On the contrary, CD patients are largely pain free (54.3% vs 41.9% in the general population, comprising n = 1071 subjects).

**Table 3:** EQ-5D-5L respondents’ self-reported health status: number and percentage by response level of patients with CD compare with general population

EQ-5D dimension	Group	Level 1	Level 2	Level 3	Level 4	Level 5	*p*^a^
		No problems	Slight problems	Moderate problems	Severe problems	Extreme problems	
		n (%)	n (%)	n (%)	n (%)	n (%)	
Mobility	CD	186 (75.3)	38 (15.4)	20 (8.1)	3 (1.2)	0 (0.0)	0.458
	GP	783 (73.1)	209 (19.5)	63 (5.9)	16 (1.5)	0 (0.0)	
Self-care	CD	235 (95.1)	10 (4.0)	0 (0.0)	2 (0.8)	0 (0.0)	0.183
	GP	992 (92.6)	60 (5.6)	16 (1.5)	3 (0.3)	0 (0.0)	
Usual activities	CD	190 (76.9)	37 (15.0)	17 (6.9)	3 (1.2)	0 (0.0)	0.420
	GP	836 (78.1)	177 (16.5)	44 (4.1)	13 (1.2)	1 (0.1)	
Pain/discomfort	CD	134 (54.3)	73 (29.6)	33 (13.4)	7 (2.8)	0 (0.0)	0.001
	GP	449 (41.9)	474 (44.3)	124 (11.6)	24 (11.6)	0 (0.0)	
Anxiety/depression	CD	162 (65.6)	70 (28.3)	15 (6.1)	0 (0.0)	0 (0.0)	0.198
	GP	663 (61.9)	310 (28.9)	75 (7.0)	15 (1.4)	8 (0.8)	

*CD* celiac disease, *GP* general population

^a^Chi-square test (comparison between CD and GP)

## Discussion

The importance of assessing the HRQoL of patients with CD has been increasingly acknowledged in research and clinical care. It complements the conventional measures of disease activity, providing better insight into the overall impact of CD on patients’ lives and their unmet needs, thereby contributing to an improved patient care and demonstrating the impact of interventions [16].

For many years, HRQoL has been widely used in the evaluation of many chronic diseases. The first studies involving HRQoL in CD were published in late 1990s [17–19], and the literature has been growing ever since. Evaluation of the QoL of CD patients has become a very important element of the diagnostic process, and minimization of the negative effects of CD on everyday functioning is a significant aim of treatment. In Slovenia, several attempts have been made to evaluate QoL in small groups of patients with CD, but those analyses were carried out only provisionally, for a specific project, and are not publicly available.

To the best of our knowledge, this is the first Slovenian study to systematically measure the HRQoL of Slovenian CD patients, by using a validated questionnaire. In this study, we used EQ-5D questionnaire to assess patients’ HRQoL. The results of our study demonstrate that CD patients have a slightly reduced HRQoL. This is in line with other studies, which have demonstrated reduced HRQol in CD patients [20, 21]. Further, a study [22] shows that HRQoL in patients diagnosed by serological screening is better than those in patients diagnosed based on clinical symptoms, and that quality of life improves substantially after diagnosis and after treatment with a GFD to levels similar to or even better than in general population [23–25]. Rare (once a year or less) visits to general practitioner were positively associated with quality of life, which is an unexpected result for a chronic disease. However, this result might demonstrate that patients have their disease under control and are following the GFD treatment. In Slovenia, the SCDS is very active, and patients can obtain all relevant information from their peers and experts involved in the SCDS, thus visits to general practitioners might be needed less frequently.

The results showed that academic education is positively associated with higher quality of life in patients. This result is not surprising as higher education is positively correlated with higher quality of life in general population as well [26–29]. Equally, evidence from population norms across countries [30] shows that quality of life decreases with age.

The mean EQ-5D scores of our study population were lower than those in the Slovenian population norms [13]. In particular, the domains on pain and mobility showed a slightly higher percentage of moderate problems in CD patients when compared to general population. The evidence demonstrates that CD patients have lowest HRQoL before the diagnosis [2, 24, 31–34] and fatigue, anxiety and depression are most obvious in patients who have not yet commenced dietary treatment [35]. Long-term impairment in quality of life has often been attributed to a lack of strict compliance with a GFD in existing studies [25, 32]. In our study, the average duration of GFD of the study participants was 16 years, and patients are active members of the SCDS, which might explain the results of low problems on the scale. It is encouraging to note that CD patients when compared with general population do not report lower overall health.

A recent review [36] showed that there was no significant difference between patients with CD and healthy controls regarding HRQoL and the results of our study are consistent with that. In the current study we measured HRQoL in already diagnosed patients. In the future, it would be good to find out if Slovenian celiac population is comparable with other studies showing that prior to diagnosis, the quality of life of people with CD is substantially lower than in the general population [23, 37]. There is still a lack of information regarding the applicability and sensitivity of these measures in determining the impact of CD on patients. To obtain more explicit information on disease-relevant areas of functioning, a well-translated and culturally adapted disease-specific HRQoL measure for CD is necessary in Slovenia. Such disease specific validated questionnaire could also enable a more accurate assessment of the effectiveness of disease management programs. Thus, for the future research on HRQoL we would recommend to include a disease specific questionnaire.

Because this was a cross sectional study, there are some limitations to this study. As data were collected once at some point, multiple factors may influence the results, as patients may complain of other systemic illnesses affecting their quality of life rather than CD. The questionnaires were sent to multiple social groups including both genders and different age groups, unfortunately, female responses (68%) were much more than male responses (32%), which might demonstrate a representation bias. The study is using a generic tool for measuring HRQoL and thus might miss certain disease specific aspects.

## Conclusion

In conclusion, our study has shown that HRQoL is slightly impaired among Slovenian patients with CD. Clinical characteristics are better determinants of their HRQoL than socio-demographic factors. Greater awareness of the impact of CD on patients’ HRQoL would improve the holistic management of CD patients.
